# Stem Cell Derived Extracellular Vesicle Therapy for Multiple Sclerosis, A Systematic Review and Meta-Analysis of Preclinical Studies

**DOI:** 10.1093/stcltm/szae011

**Published:** 2024-03-20

**Authors:** Mehri Barabadi, Madison C B Paton, Naveen Kumar, Rebecca Lim, Natalie L Payne

**Affiliations:** The Ritchie Centre, Hudson Institute of Medical Research, Victoria, Australia; Department of Obstetrics and Gynaecology, Monash University, Victoria, Australia; Cerebral Palsy Alliance Research Institute, Speciality of Child and Adolescent Health, Sydney Medical School, Faculty of Medicine and Health, The University of Sydney, New South Wales, Australia; Department of Paediatrics, Monash University, Victoria, Australia; The Ritchie Centre, Hudson Institute of Medical Research, Victoria, Australia; Department of Obstetrics and Gynaecology, Monash University, Victoria, Australia; The Ritchie Centre, Hudson Institute of Medical Research, Victoria, Australia; Department of Obstetrics and Gynaecology, Monash University, Victoria, Australia; Australian Regenerative Medicine Institute, Monash University, Victoria, Australia

## Abstract

Stem cell therapy holds promise for multiple sclerosis (MS), with efficacy of different stem cell types reported across a range of preclinical MS animal models. While stem cell therapy has been approved for a small number of diseases in humans, extracellular vesicles (EVs) may provide an efficacious, cost-effective, and safer alternative to stem cell therapy. To this end, we conducted a systematic review with meta-analysis to assess the effectiveness of stem cell-derived secretome (EV and conditioned media (CM)) in animal models of MS. The data were extracted to calculate standardized mean differences for primary outcome measure of disease severity, using a random effect model. Additionally, several subgroup analyses were conducted to assess the impact of various study variables such as stem cell type and source, stem cell modification, route and time of administration, number of animals and animal’s age, and EV isolation methods on secondary outcome. Publication quality and risk of bias were assessed. Overall, 19 preclinical studies were included in the meta-analysis where stem cell EV/CM was found to significantly reduce disease severity in EV-treated (SMD = 2, 95% CI: 1.18-2.83, *P* < .00001) and CM-treated animals (SMD = 2.58, 95% CI: 1.34-3.83, *P* < .00001) compared with controls. Our analysis indicated that stem cell secretome has a positive effect on reducing demyelination, systemic neuroinflammation, and disease severity in preclinical models of MS. These findings indicate a potential therapeutic effect that merits investigation and validation in clinical settings.

**Significance Statement**

Multiple sclerosis (MS) is an immune-mediated disease of the central nervous system resulting in progressive neurological, cognitive, and physical disability. Stem cells provide immunomodulatory and neuroprotective effects and may reduce the progression of disease in MS, however their secretome products (including EVs/CM) while thoroughly assessed in pre-clinical models, have yet to be translated to the clinic.

This systematic review and meta-analysis assessed the therapeutic benefits of stem cell-derived EV and CM on preclinical models of MS, showing a significant therapeutic benefit that should be further explored in clinical settings. Importantly, this study highlights methodological gaps in reporting study outcomes and the need to utilize existing guidelines to facilitate the clinical translation of this newly emerging therapy for MS.

## Introduction

Multiple sclerosis (MS) is a neuroinflammatory and neurodegenerative autoimmune disease of the central nervous system (CNS) affecting approximately 2.8 million people worldwide.^1^ Pathological hallmarks include multifocal demyelinating lesions, astrogliosis, and neuronal degeneration.^2^ Current treatments targeting neuroinflammation have little to no effect on promoting myelin or neuronal repair and are associated with considerable side effects.^3^

Stem cells show great promise as a treatment for MS based on mounting preclinical data^4^ and clinical trial results.^5-7^ The beneficial effects of stem cells, either infused into the bloodstream or transplanted directly into the CNS, have largely been attributed to their secretome, which includes soluble factors and extracellular vesicles (EVs).^8^ Stem cell-derived EVs may offer an improved safety profile, including limited immunogenicity, along with enhanced neuroprotective potential, as they are small enough to cross the blood–brain barrier and reach the CNS.^9^

Here, we conducted a meta-analysis of studies assessing the efficacy of the stem cell-derived secretome, encompassing both stem cell conditioned media (CM) and EVs, in preclinical animal models of MS. We evaluated the effect of study design variables such as cell origin and type, disease model, delivery route and dose on the primary outcome measure of disease severity and the secondary outcome measures of CNS pathology and repair, and inflammatory response. The results of this study will help to identify limitations in the current preclinical study design and set a foundation for forthcoming clinical trials investigating stem cell-free therapies for MS.

## Methods

This study was designed and performed in accordance with the guidelines of Preferred Reporting Items for Systematic Reviews and Meta-Analyses (PRISMA, http://www.prisma-statement.org/).^9^ The review protocol was developed and registered on PROSPERO (CRD42021273546).

### Selection Criteria

Preclinical studies, published in English, were included if they assessed the efficacy of stem cell CM or EVs in an animal model of MS. Studies that did not include an untreated control group or state the number of animals in the experiment groups were excluded. EVs could be given at any time before or after induction of disease and via any route of administration. Eligible studies must have included primary outcome measures of disease severity and at least one of the following categories of secondary outcome measures: Quantitative analysis of CNS pathology, CNS repair or inflammatory response. Studies that failed to meet these criteria were excluded.

### Search Strategy

A comprehensive literature search was performed to identify publications in Ovid MEDLINE(R), Ovid EMBASE, Web of Science, PubMed, and Scopus databases from 1946 to September 2022. The search strategy was designed by a medical reference librarian and conducted by the authors. The search strategy, including all the search terms, is available in Supplementary File 1. To ensure no recent studies were missed, searches were re-run using the same parameters until June 2023.

### Study Selection Process

**Figure 1.**
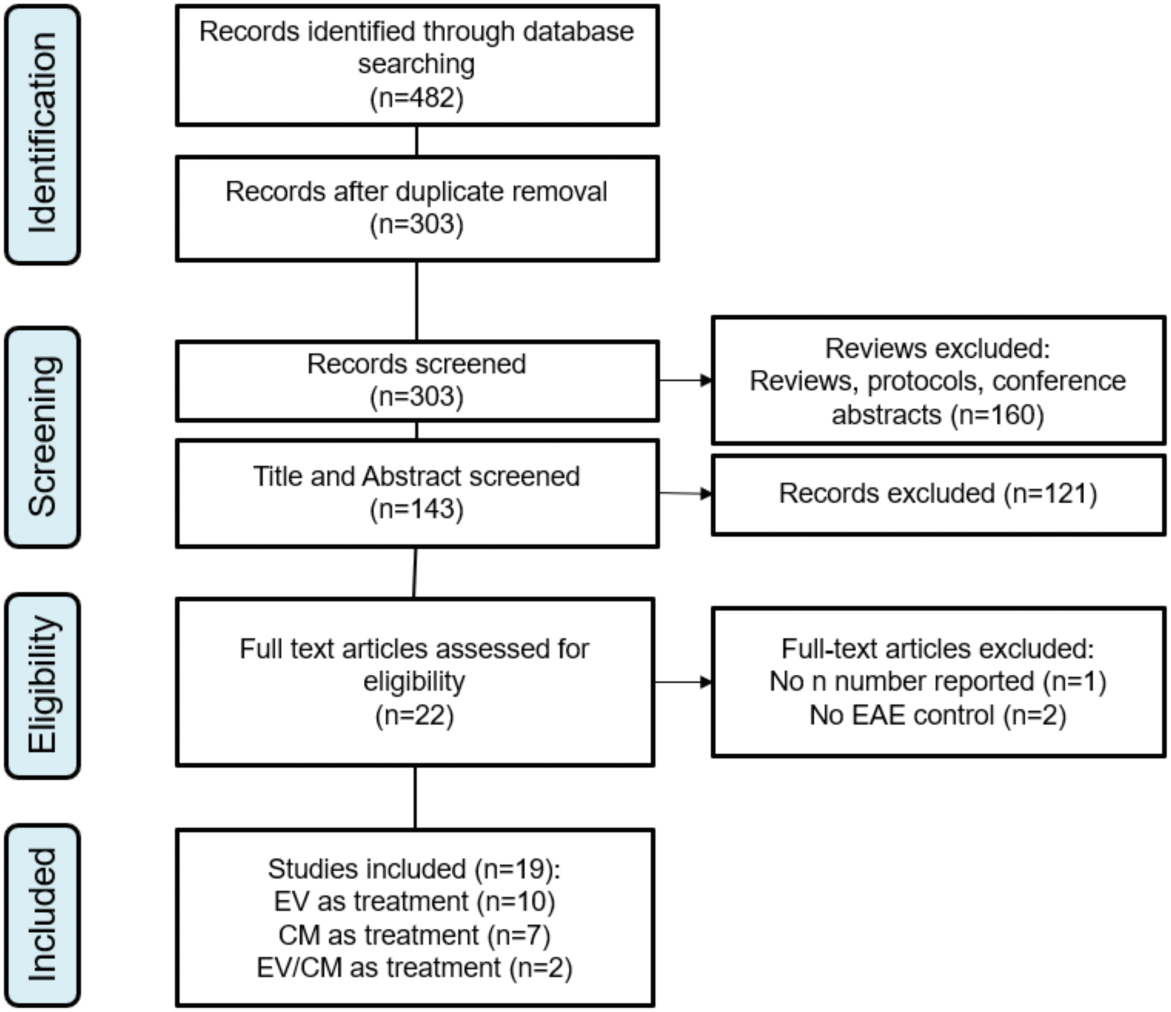
Flow diagram of the study selection process.

Figure 1 depicts the study selection process. First, search results were exported into Mendeley (version 1.19.8) to remove duplicates, then manually checked by reviewers to confirm correct deduplication. Titles of publications were then screened to exclude reviews, protocols, and abstracts before identifying the studies that met inclusion criteria. The full text of eligible studies was retrieved and independently assessed by 2 reviewers, and any disagreements were resolved by a third reviewer.

### Data Extraction

To conduct a meta-analysis for primary and secondary outcomes, study design features such as the number of animals used, the experimental methods, and quantitative data, were extracted from eligible studies via 2 independent review authors. Additionally, details of interventions including the animal age, EAE scoring system, stem cell type and source, stem cell modification, EV route and time of administration, and EV isolation methods were extracted to investigate the effect of such variables on the primary outcome using subgroup analysis. In case of discrepancies in extracted data, a third reviewer was involved. Authors of included studies were contacted twice if relevant data were unavailable in the published manuscript or supplementary materials. If no response was received, the data were excluded.

### Quality Assessment

To estimate the risk of bias (RoB), we used the Systematic Review Centre for Laboratory Animal Experimentation (SYRCLE) risk of bias assessment tool.^10^ Two reviewers independently judged the quality of each included paper to assess the selection bias, performance bias, detection bias, attribution bias, and reporting bias, where a “Yes’’ score indicates low risk; a “No” indicates high risk, and a “?” indicates an unknown risk of bias. Furthermore, we added 3 more items to the RoB assessment according to the consensus statement “good laboratory practice in the modeling of stroke,^11^” including sample size calculation, statement of any potential conflict of interest and reporting of animal welfare, with conflict of interest determined through funding statements or author declaration. Furthermore, we expanded the RoB assessment to include 2 additional criteria aimed at recognizing efforts to mitigate bias in cases where randomization and blinding were only partially reported and received an unclear score in the SYRCLE assessment. In case of any disagreements, one additional author was involved.

### Data Analysis

Review Manager version 5.4 was used to conduct a quantitative analysis of outcomes. We used a random-effects inverse variance model to evaluate the Hedges’s g standardized mean difference (SMD) and 95% confidence interval (CI) for all continuous data. The SMD was selected over raw mean difference to count for the different scales used to measure the same outcome. The effect of heterogeneity was assessed using the *I*^2^ statistic, with values of 0%-25%, 25%-50%, 50%-75%, and >75% considered to be very low, low, moderate, and considerable heterogeneity, respectively. Subgroup analysis was performed where sufficient data were available (2 or more studies in each subgroup) to determine potential sources of heterogeneity. Where the studies included 2 or more treatment groups, each treatment group was counted as a separate study. In the studies where the standard error of the mean was reported, the standard deviation was derived from the standard error of the mean and *n* numbers. Subcategorised studies for meta-analysis have been listed in Supplementry Table S7.

## Results

### Study Selection

A total of 482 records were identified following the search procedure shown in Fig. 1. After excluding duplicates, 303 studies were screened to exclude reviews, protocols, and conference abstracts and 143 studies were screened by title and abstract. Full-text screening for eligibility was performed on 22 studies and based on the inclusion criteria, 19 studies were included in the meta-analysis ranging from 2016 to 2023.

### Characteristics of Included Studies

**Figure 2.**
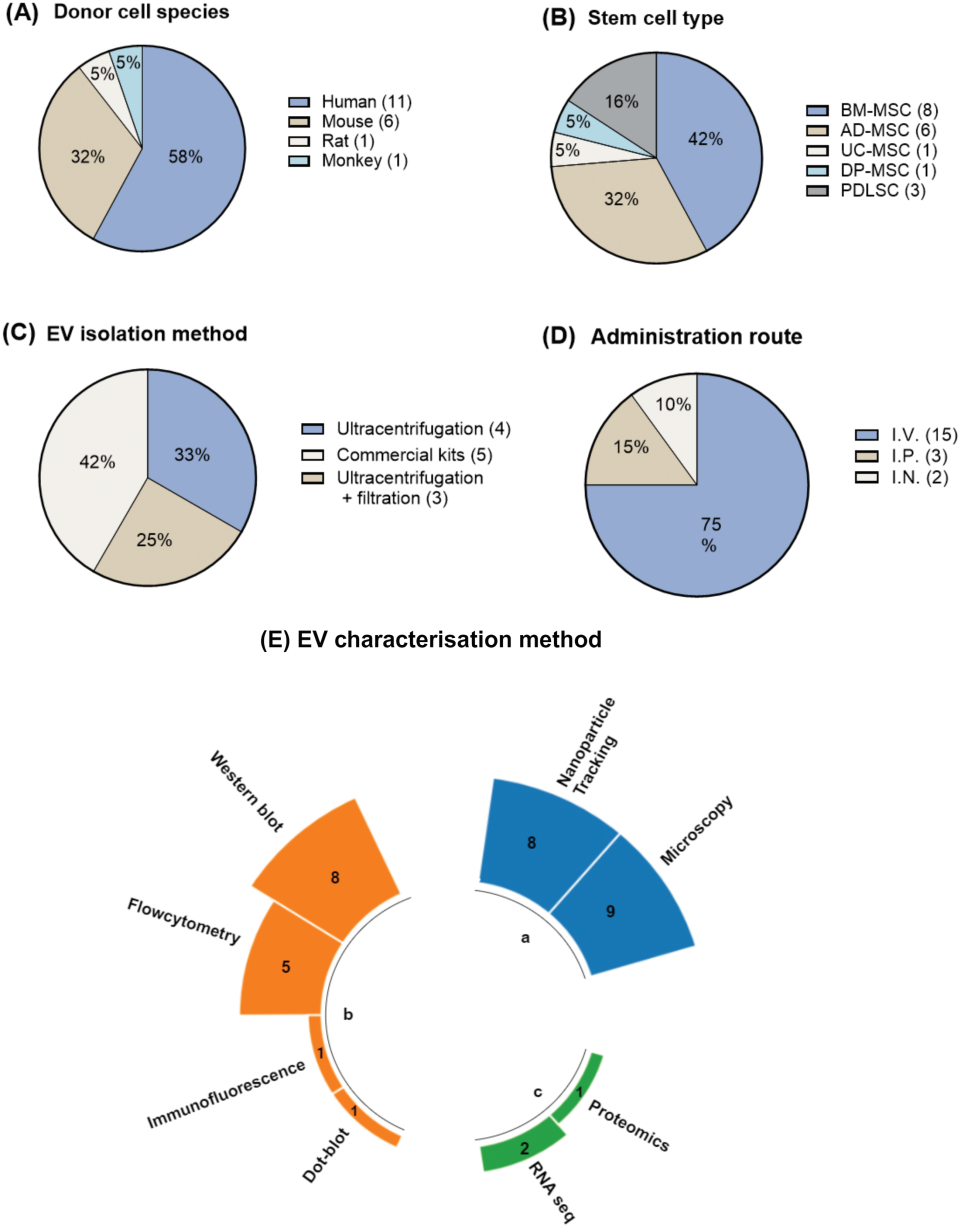
An overview of characteristics of included studies in meta-analysis. (**A**) The sources of the stem cells used; (**B**) stem cell types; (**C**) EV isolation methods; (**D**) administration route; (**E**) summary of EV characterization methods used in the included studies based on the morphology and size (a), EV markers (**b**) and the RNA and protein cargo (**c**). I.V. intravenous; I.P. intraperitoneal; I.N. intranasal.

The characteristics of animal models and therapeutic interventions are summarized in Table 1 and Supplementary Table S1. All studies were performed in mice (*n* = 17, 89%) or rats (*n* = 2, 11%). The MS models used were experimental autoimmune encephalomyelitis (EAE) (*n* = 18), Theiler’s murine encephalomyelitis virus (TMEV) induced demyelination model (*n* = 1), cuprizone induced demyelination model (*n* = 1) or lysolecithin (LPC)-induced demyelination model (*n* = 1). All EAE studies used MOG_35-55_ as the immunizing antigen, with one study using both MOG_35-55_ and recombinant (r)MOG (Table 1). The majority of studies used human cells as the cell source (*n* = 11, 58%) (Fig. 2A) and mesenchymal stem cells (MSCs) were the most common cell type used to isolate EV or CM (84%, *n* = 16) (Fig. 2B). Intravenous injection was the most common administration route (*n* = 15, 75%) (Fig. 2D) and commercial EV isolation kits were the most common isolation method used to isolate EVs (*n* = 5, 42%) (Fig. 2C).

**Table 1.** Characteristics of animal models used in included studies.

Study	Year	Species			Animal model
		Strain	Sex	Age (weeks)	
Riazifar^12^	2019	C57Bl/6	Female	6-8	EAE
Fathollahi^13^	2020	C57Bl/6	Female	6-8	EAE
Shamili^14^	2018	C57Bl/6	Female	10-13	EAE
Li^15^	2018	SD rat	Female	Not reported	EAE
Koohsari^16^	2021	C57Bl/6	Female	6-8	EAE
Farinazzo^17^	2018	C57Bl/6	Not reported	6-8	EAE
Laso-García^18^	2018	SJL/J	Female	4-6	TMEV
Jafarinia^19^	2020	C57Bl/6	Female	6-8	EAE
Rajan (a)^20^	2016	C57Bl/6	Male	12	EAE
Rajan (b)^21^	2017	C57Bl/6	Male	12	EAE
Giunti^22^	2021	C57Bl/6	Female	6-8	EAE
Zhang Jing^23^	2022	C57Bl/6	Female Male	6-8 8	EAE CPZ
Bai^24^	2012	C57Bl/6	Female	8-12	EAE
Wang Xi^14^	2019	C57Bl/6	Female	Not reported	EAE
Giacoppo^25^	2017	C57Bl/6	Female	6-7	EAE
Yousefi^26^	2016	C57Bl/6	Female	6-7	EAE
Sargent^27^	2017	C57Bl/6	Female	10-12	EAE
Shimojima^28^	2016	C57Bl/6	Female	8	EAE
Galeshi^29^	2019	Wistar Rat	Male	Not reported	LPC

EAE, experimental autoimmune encephalomyelitis; SD, Sprague Dawley; TMEV, Theiler’s murine encephalomyelitis virus.

In terms of EV characterization methods (Fig. 2E), electron microscopy was the most commonly used technique to study size and morphology (*n* = 8, 67%). Western blot was the most common technique used to assess EV marker expression (*n* = 7, 58%). Only one out of 12 studies with EV treatment conducted RNA sequencing to assess RNA cargo, and 2 conducted proteomic analysis to assess protein cargo. Expression of CD63 (*n* = 10, 83%) and CD9 (*n* = 7, 58%) were the most common markers used for EV characterization. A detailed list of EV characterization methods for each included study is provided in Supplementary Table S2.

### Meta-Analysis of Primary Outcome

#### Extracellular Vesicles (EVs)

**Figure 3.**
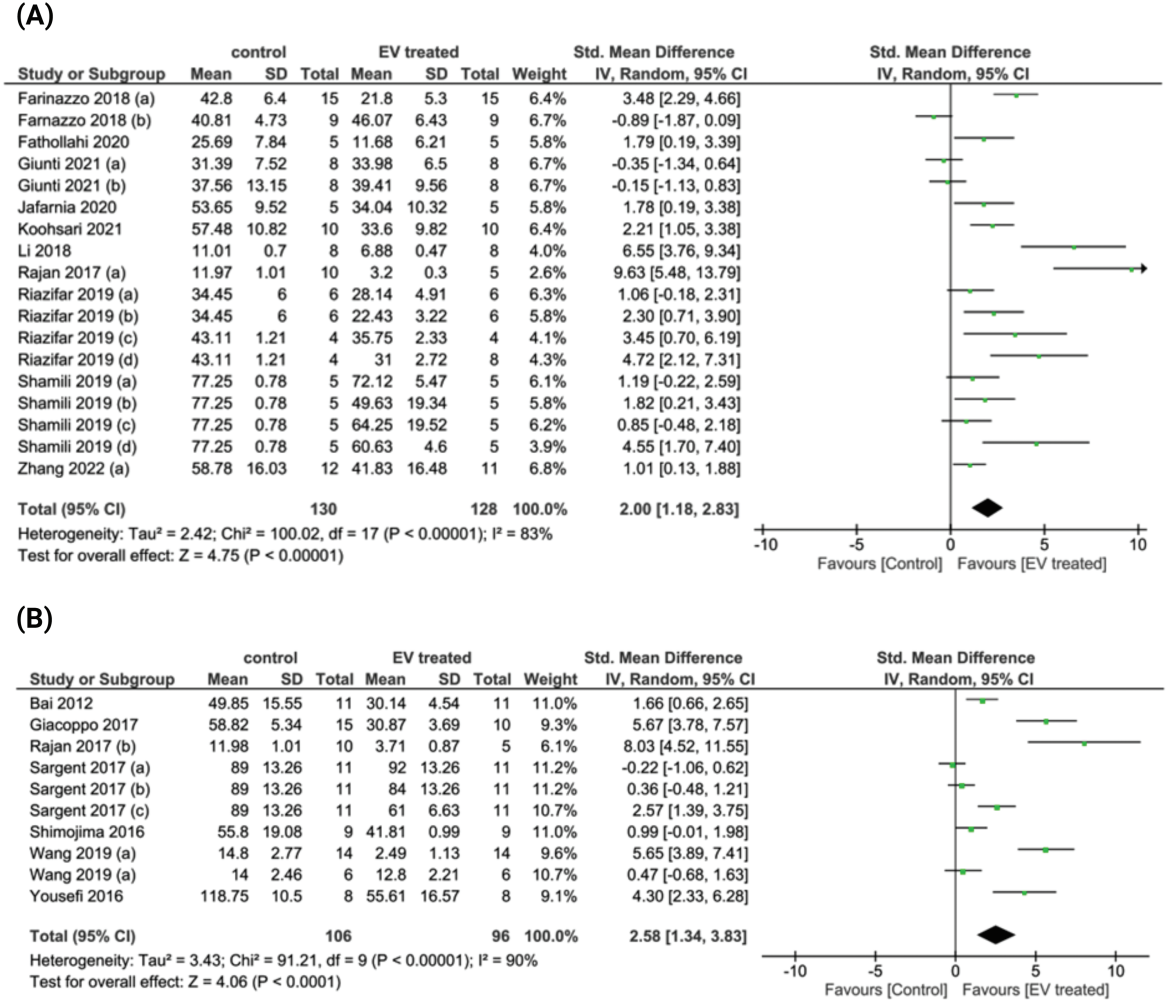
Forest plots of the standardized mean differences (SMDs) demonstrate the effect of stem cell-derived EVs (**A**) and CM (**B**) on the cumulative disease score. The random-effects consistency model estimated the SMDs (with 95% credible intervals).

The impact of stem cell-derived EVs on disease severity in studies utilizing the EAE model (*n* = 11) was analyzed using the cumulative disease score, defined as the sum of the daily clinical scores throughout the EAE observation period. EV treatment significantly reduced disease severity compared to controls with an SMD of 2.00, 95% confidence interval (CI) (1.18-2.83, *P* < .00001; high heterogeneity *I*^2^ = %83; Fig. 3A). To investigate potential sources of heterogeneity, subgroup analysis was conducted for administration route (2 subgroups: intravenous and other routes), stem cell source (4 subgroups: BM-MSC, AD-MSC, UC-MSC, and PDLSC), stem cell species (3 subgroups: human, mouse/rat, and monkey), animal age (2 subgroups; 6-8 weeks old and older than 8 weeks old), EAE scoring system (4 subgroups: 0-10, 0-7, 0-5, and 0-4), treatment paradigm (2 subgroups: prior to onset of clinical disease or prophylactic and after disease onset or therapeutic), EV isolation method (3 subgroups: ultracentrifugation, a combination of ultracentrifugation and ultrfiltration, and commercial kits), and EV modification (2 subgroups: naïve EVs and primed/engineered EVs) (Table 2).

**Table 2.** Subgroup meta-analysis stem cell-derived EV treatment for the primary outcome of disease severity score.

Factor	SMD (95% CI)	*I* ^2^ (%)	Heterogeneity *P* value	Effect size *P* value	df	Subgroup difference *P* value
Stem cell species						
Human (*n* = 7)	2.86 (1.59, 4.13)	70	.003	<.0001	6	.06
Mouse/rat (*n* = 9)	1.59 (0.42,2.76)	86	<.00001	.008	9	
Monkey (*n* = 2)	1.01(0.13,1.88)	–	–	.48	–	
Stem cell type						
BM-MSC (*n* = 12)	1.79 (0.90, 2.68)	77	<.00001	<.0001	11	.004
AD-MSC (*n* = 4)	1.52 (−0.63,3.66)	91	<.00001	.17	3	
UC-MSC (*n* = 1)	2.21 (1.05,3.38)	–	.0002	.0002	–	
PDLSC (*n* = 1)	9.63 (5.48,13.79)	–	<.00001	<.00001	–	
EV isolation method						
Ultracentrifugation (*n* = 6)	2.99 [1.52, 4.45]	71	.004	<.00001	5	.29
Ultracentrifugation (*n* = 4) and ultrafiltration	1.43 [−0.34, 3.20]	91	<.00001	.11	3	
Commercial kits (*n* = 8)	1.64 (0.42, 2.86)	81	<.00001	.008	7	
Animal’s age (weeks)						
6-8 (n = 12)	1.49 (0.60, 2.38)	83	<.00001	.001	11	.81
>8 (*n* = 4)	1.67 (0.52, 2.81)	47	.13	.004	3	
EAE scoring system						
0-4 (*n* = 4)	2.55 (1.03, 2.83)	60	.06	<.00001	3	.002
0-5 (*n* = 7)	1.35 (-0.02, 2.71)	90	<.00001	<.00001	6	
0-7 (*n* = 6)	1.75 (1.03, 2.47)	25	.24	.05	5	
0-10 (*n* = 1)	9.36 (5.48,13.79)	-	<.00001	.001	-	
Route of administration						
Intravenous (*n* = 15)	2.31 (1.24, 3.38)	87	<.00001	<.0001	14	.15
Other (*n* = 2)	0.71 (-1.18, 2.6)	76	.04	.46	1	
Treatment paradigm						
Prophylactic (*n* = 4)	3.60 (1.38, 5.82)	83	.0005	.0002	3	.09
Therapeutic (*n* = 14)	1.55 (0.73, 2.37)	80	<.00001	.001	13	
EV modification (primed/engineered)						
Naïve (*n* = 13)	1.91 (0.93, 2.89)	85	<.00001	.0001	12	.66
Engineered/primed (*ns* = 5)	2.37 (0.56, 4.19)	82	.0002	.01	4	

#### Conditioned Media

CM treatment in the EAE model conferred a large effect size with an SMD of 2.58CI (1.34 and 3.83, *P* < .0001; high heterogeneity *I*^2^ = %90; Fig. 3B). The small number of studies utilizing CM treatment prevented further subgroup analysis.

### Subgroup Analysis of Primary Outcome for EV Treatment

Subgroup analysis of different parameters was conducted for stem cell-derived EV treatment to further assess the source of heterogeneity in the primary outcome measure of disease severity (Table 2). The subgroup analysis showed no significant difference in the treatment effect size based on EV isolation and modification methods (*P* = .29 and *P* = .66 respectively), the age of animals (*P* = .81), or route of administration (*P* = .15).

A significant effect size was observed when comparing the EAE scoring systems (*P* = .002). The stem cell species and stem cell type were another source of heterogeneity, with human stem cells showing the highest effect size compared to the other groups (*P* < .00001). However, these data should be treated with caution due to the low number of studies in some of the subgroups.

### Meta-Analysis of Secondary Outcomes

The secondary outcomes of the included studies are summarized in Supplementary Table S3. These outcomes were subcategorized into CNS pathology, CNS repair, and inflammatory response. No meta-analysis was performed for subgroups with an insufficient number of studies using the same analysis method.

### Effect of Stem Cell-Derived EVs and CM on CNS Pathology

#### CNS Inflammation

**Figure 4.**
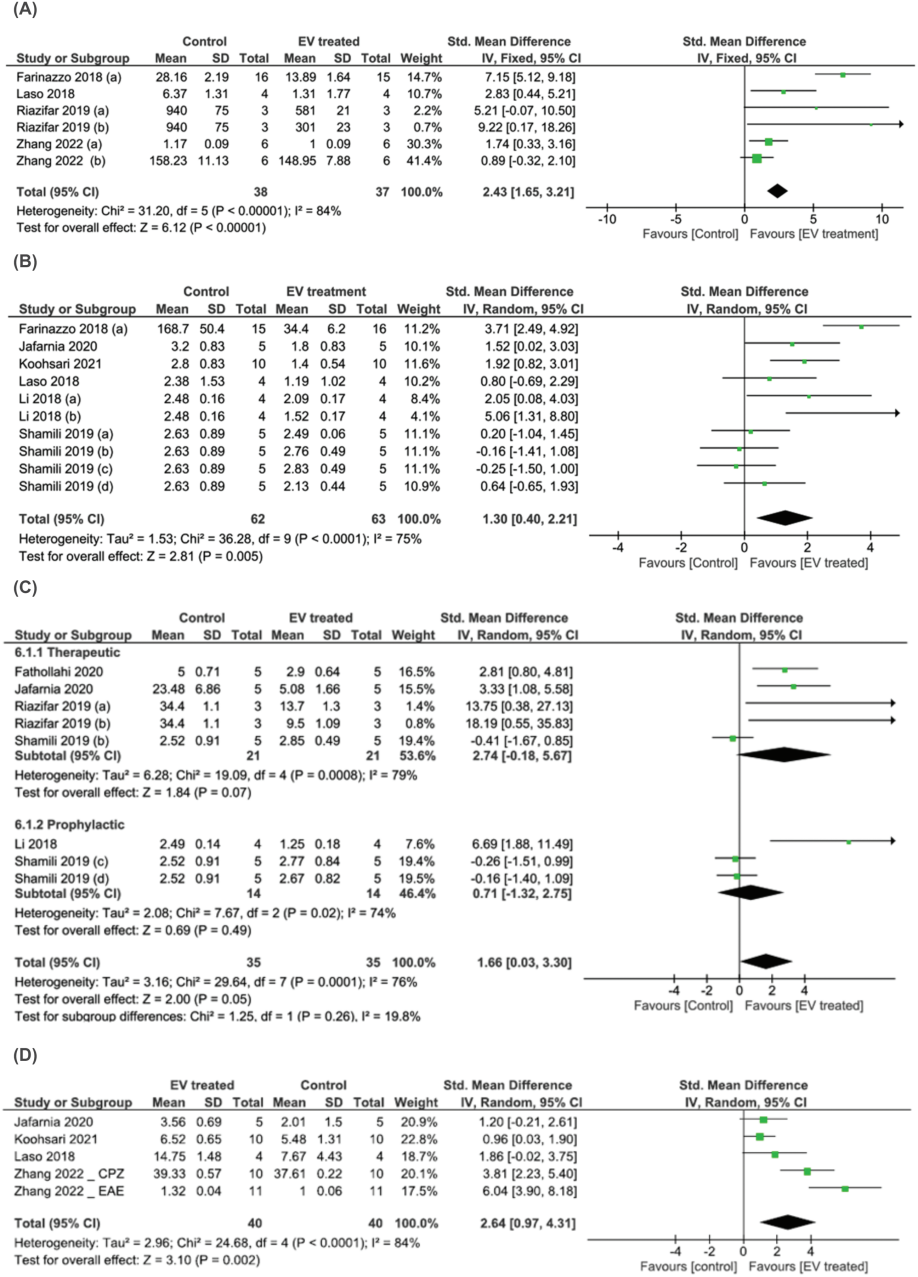
Forest plots of the standardized mean differences (SMDs) demonstrating the effect of stem cell-derived EVs on CSN inflammation and demyelination. (**A**) Reduction in the percentage of microglia in CNS measured by Iba-1 staining and (**B**) in immune cell infiltration into CNS measured by H&E staining was observed in EV-treated studies. (**C**) Reduction in the percentage of the demyelinated area measured by LFB staining and (**D**) higher MBP staining was observed in the mice receiving EV therapeutically. The random-effects consistency model estimated the SMDs (with 95% credible intervals).

CNS inflammation was assessed by quantifying either the number of microglia and/or macrophages (Iba-1, CD68, YM1, CD86, iNOS, Arg1, and CD206) or infiltrating leukocytes (CD3, CD4, and CD45) or through semi-quantitative analysis of H&E) stained sections. The analysis of Iba-1 staining in the EV-treated studies showed a significant reduction in the number of microglia in the CNS after EV administration (SMD = 2.43, 95% CI, 1.65-3.21, *P* < .0001) (Fig. 4A). Likewise, H&E staining showed a significant reduction in immune cell infiltration into CNS after EV administration (SMD = −1.3, 95% CI: −2.21 to −0.4, *P* < .0001) (Fig. 4B).

#### Demyelination

The effect of stem cell-derived EV treatment on demyelination was measured using different methods, with some studies utilizing multiple methods of analysis (Supplementary Table S3). Luxol fast blue (LFB) staining was used to measure the percentage of demyelinated area in 28% (*n* = 5) of the EAE studies, with one study not reporting any quantitative data. Overall, LFB staining showed that EV-treatment significantly reduced demyelination in the CNS of EAE mice (SMD = 1.66, 95% CI: 0.03–3.30, *P* = .0001) (Fig. 4C). This was due to the effect size of the therapeutic treatment approach (SMD = 2.74, 95% CI: −0.18 to 5.67, *P* = .00008), as no significant impact on demyelination was shown when only prophylactic administration of EVs were assessed (SMD = 0.71, 95% CI: −1.32 to 2.75, *P* = .02).

Myelin basic protein (MBP) staining was used in 4 studies with a therapeutic treatment approach. Mice receiving EVs therapeutically showed significantly higher levels of MBP staining in the CNS compared to controls (SMD = −2.64, 95% CI: −0.97 to −4.31, *P* < .0001) (Fig. 4D).

#### Axonal Damage

The effect of EV or CM treatment on axonal damage is presented in Supplementary Table S3. Axonal injury was quantified in only 28% (*n* = 5) of the EAE studies included in the meta-analysis and notably, each study used a different technique for their analysis. The methods used in the EV studies were Bielschowsky silver and toluidine blue staining (*n* = 1), amyloid-beta precursor protein staining (APP) (*n* = 1), and Golgi (*n* = 1) staining.

In the study utilizing Bielschowsky and Toluidine blue staining techniques, while quantitative data were lacking for Bielschowsky silver staining, morphometric measurements of Toluidine blue staining of the spinal cord showed that both naïve and engineered MSC-EV treatments elicited changes in the local fiber count and the thickness of myelin sheaths (*G*-ratios). In the study utilizing APP staining, MSC-EV treatment significantly reduced APP + density in the spinal cord, a commonly used marker for evaluating axonal damage in the CNS. Furthermore, when Golgi staining was applied to EAE mice to quantify dendritic spine numbers, both hPDLSCs-CM and EVs extracted from RR-MS patients exhibited a significant increase in spine density. In the sole study investigating CM use in the EAE model, utilizing SMI31 (*n* = 1) staining, a substantial decrease in axonal injury was observed.

#### Astrogliosis

Glial fibrillary acidic protein staining (GFAP), a histological method to detect glial cells, was reported in 3 studies. One study reported no effect on GFAP staining in the EV treated group compared to the control in an EAE model. The second study, using EVs in a TMEV model of MS, reported a significant reduction in GFAP staining in the brain but no significant difference in the spinal cord. The third study using CM in a LPC demyelination model reported a significant reduction in GFAP staining in the brain.

### Effect of Stem Cell-Derived EVs on CNS Repair

The effect of EV treatment on remyelination and repair was evaluated in 2 demyelination models. In EAE studies (*n* = 4) oligodendrocyte progenitor cell (OPCs) surface markers such as NG2 (*n* = 2), Olig2 (*n* = 1), APC (*n* = 1) was measured, or *G* ratio (*n* = 1) was calculated. Of all these studies, 3 studies reported a significant increase in the number of OPCs after EV treatment. In CPZ studies (*n* = 1), a significant increase in APC was observed.

### Effect of Stem Cell-Derived EVs on Inflammatory Response

#### Cytokines

The effect of EV treatment on the cytokine profile in the serum, spleen, and CNS are summarized in Supplementary Table S3. Cytokine results were highly variable, and studies vary significantly in their approaches to examining these cytokine responses. While cytokines are not consistently measured within the same organ or biological sample, there is a general trend to compare levels of inflammatory and anti-inflammatory cytokines.

#### Regulatory T Cells

**Figure 5.**
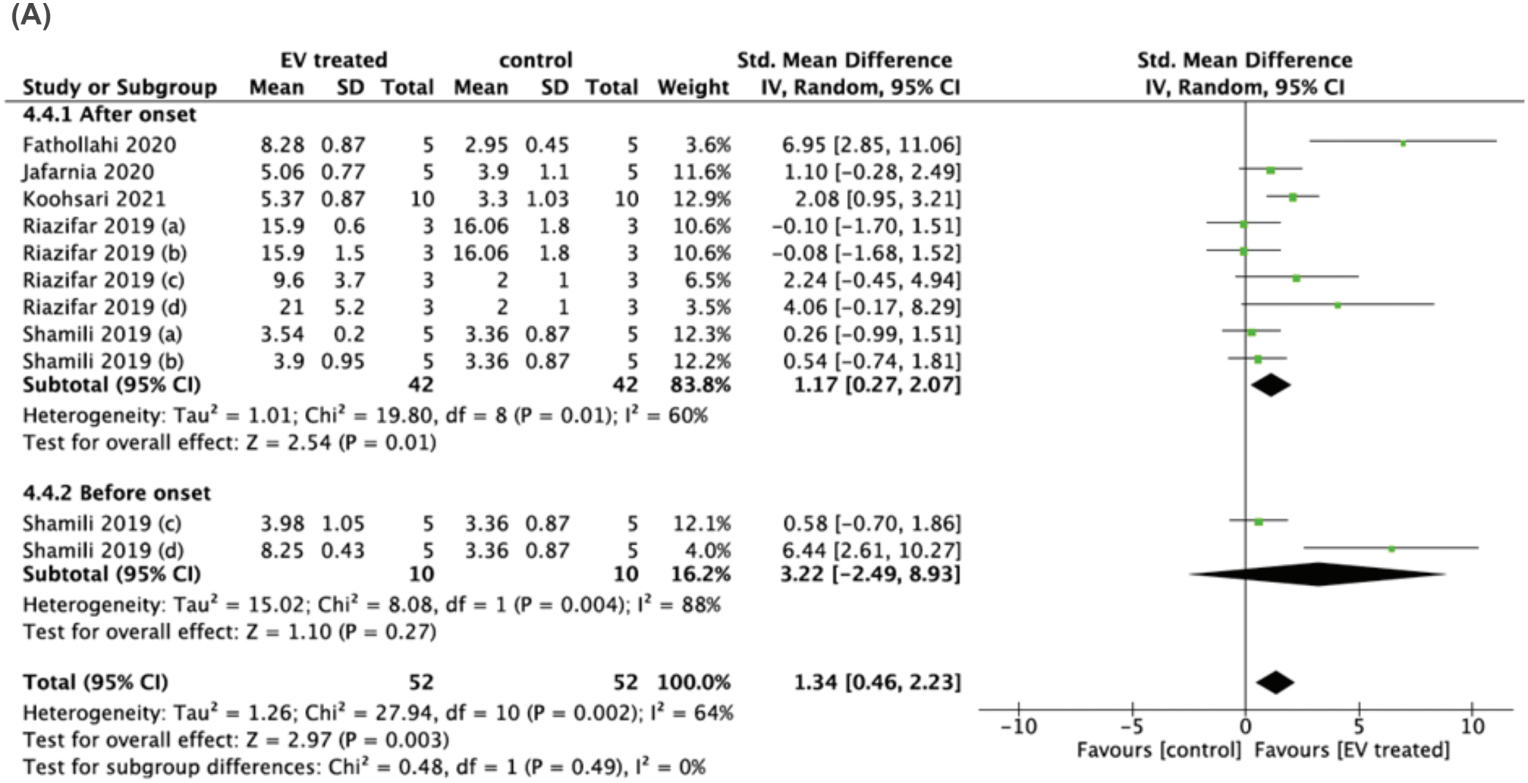
Forest plots of the standardised mean differences (SMDs) demonstrate the effect of stem cell-derived EVs administered after or before disease onset on regulatory T cells in the spleen. The random-effects consistency model estimated the SMDs (with 95% credible intervals).

EV treatment significantly increased regulatory T cells (Tregs) in the spleen (SMD = 1.34, 95% CI: 0.46-2.23, *P* = .002) (Fig. 5). Time of administration had an impact on the effect size, with the prophylactic approach (EV treatment before disease onset) (SMD = 3.22, 95% CI: −2.49 to 8.93, *P* = 0.27) having a higher effect on the quantity of Tregs compared to the therapeutic approach (SMD = 1.18, 95% CI: 0.14-2.22, *P* = .03).

### Study Quality and Risk of Bias Assessment

**Figure 6.**
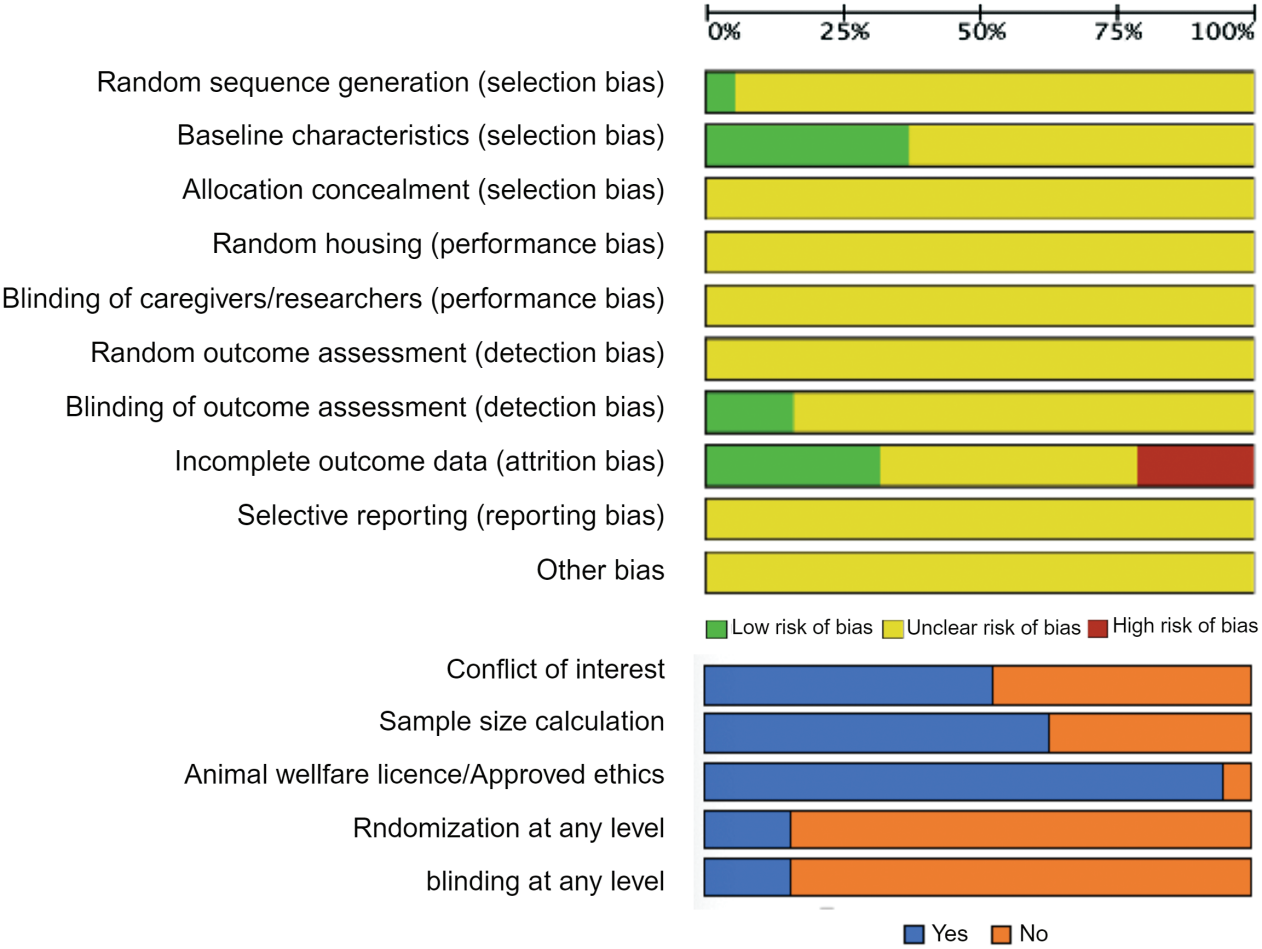
Risk of bias of the included studies. The CYRCLE risk of bias items (first 10 items) evaluated the quality of included studies by reporting “Yes” for low risk of bias, “No” for high risk of bias, and “Unclear” if not reported.

The risk of bias assessment from the 19 studies is presented in Fig. 6. While 6 of the studies (37%) reported randomization of animals after EAE induction and before treatment administration, 21% of the studies reported randomization before EAE induction (Supplementary Table S4). One study reported randomization before and after EAE induction, and the remaining studies did not mention any randomization. The randomization method (ie, sequence generation) was mentioned in only one publication and none of the included studies described their allocation concealment methods. Partial outcome assessment blinding was reported in 32% of publications, while only 3 studies documented blind outcome assessment for all outcomes.

While only 2 studies reported the mortality rate, a high risk of attrition bias was identified in 21% of the publications due to discrepancies between the number of animals registered in the methods and results sections without any reports of animal dropout. Sample size calculation was reported in 16% (*n* = 3) of the studies and animal welfare licence or approved ethics was mentioned in 95% of the studies. The funding source was reported in 69% of the included studies and 74% of the studies reported no conflict of interest. One study reported an industry source of funding, and 2 studies did not report any funding source or whether conflicts of interest existed. A detailed scoring for each included study is provided in Supplementary Table S4 and Supplementary Fig. S1.

## Discussion

To obtain an overview of the therapeutic efficacy of stem cell-free therapy in MS, we systematically reviewed preclinical studies of MS treating with stem cell-derived CM or EVs. Out of the 22 included studies, with 19 eligible for meta-analysis, all were performed in small animal models and the majority used the EAE model of MS. We found that stem cell-derived EVs in preclinical models of MS resulted in a statistically significant treatment effect on disease severity and secondary outcomes, including CNS pathology and repair and inflammation. Similarly, stem cell-derived CM showed significant therapeutic benefits on disease severity.

We also found variations in the EAE immunization protocol (Supplementary Table S5), which can directly affect the severity and immunopathogenesis of the disease. In our meta-analysis, 94% (*n* = 16) of EAE studies used MOG peptide to induce the disease with different amounts of MOG and *Mycobacterium tuberculosis*. This variability can lead to different levels of activation of the innate immune system^30^ and disease manifestations, with high levels of MOG causing a chronic, non-remitting disease model and low levels of MOG inducing a relapsing-remitting disease course.^31^ Additionally, it is known that environmental factors can influence the development of EAE, indicating that the same dose of EAE induction reagents will not produce the same condition across all facilities.^32^

We observed heterogeneity in stem cell species and types. It is essential to highlight the limited studies involving monkeys (*n* = 1) compared to the larger sample size in human studies (*n* = 7) and mouse/rat models (*n* = 9). Similar concerns arise due to varying sample sizes among different stem cell types—such as BM-MSC (*n* = 12), AD-MSC (*n* = 4), UC-MSC (*n* = 1), and PDLSC (*n* = 1). These differences strongly suggest a potential influence on the observed heterogeneity in stem cell species and types. Further analysis and discussion, particularly focusing on the impact of sample size variations across species and stem cell types, could provide insights into the reasons behind this observed diversity.

The subgroup analysis of disease severity also showed heterogeneity in the time of administration. Of note, the time of treatment administration significantly impacted disease severity and secondary outcomes such as demyelination and inflammation. This finding supports the potential benefits of early intervention in the treatment of MS in a preclinical setting.^23^ The highly dynamic pathophysiological response in MS involves the recruitment of innate and adaptive immune cell types to the CNS. Different pathological mechanisms are observed between patients and at different stages in the disease course, along with significant heterogeneity in terms of clinical presentation.^33,34^While our findings highlight the benefits of earlier intervention in improving disease severity outcomes, a limitation of this meta-analysis is that all the EAE studies used the CD4^+^ T-cell-mediated MOG-EAE model in C57BL/6 mice. Other strains and methods for EAE disease induction are available that recapitulate a broader spectrum of clinical courses and mechanisms of pathogenesis observed in MS.^35^

Our analysis of the secondary outcome measures was limited due to the small number of included studies, as well as the diverse range of approaches used to quantify these changes. Of note, only 2 of the included studies used toxin-induced MS animal models, which allow for the study of remyelination in the absence of strong peripheral immune cell activation. The ability of EVs/CM to promote neuroprotection and enhance CNS remyelination, which are crucial for limiting progression of neurodegeneration in MS, still requires more detailed assessment in such models prior to clinical translation due to their incomplete replication of all aspects of MS.

### EV Treatment

A diverse range of methods was used to identify the RNA and protein cargo of EVs and to characterize their surface and internal markers. Based on minimal information for studies of extracellular vesicles (MISEV) guidelines for the protein content-based EV characterization, 5 categories of proteins are required for a thorough description. Tissue-specific/nonspecific tetraspanins, cytosolic proteins, and components of non-EV co-isolated structures are the first 3 categories that are aimed at determining EV nature and purity. The other 2 categories including proteins associated with other intracellular compartments than endosomes and secreted proteins recovered with EVs were required if the study asked questions specific to intracellular localization and biological function.^36^ In our meta-analysis, only one study included all the proposed protein categories by MISEV, and 70% (*n* = 9) of studies only characterized proteins from category one (Supplementary Table S2).

The storage temperature for EVs and CM was not reported consistently, with only 26% (*n* = 6) of the included studies providing details. Of those studies, 3 reported -8 0°C and the others reported −70 °C, −20 °C, and 4 °C as the storage temperature of EVs or CM. We previously discussed the challenges associated with the storage medium, storage temperature, and the effect of freeze-thaw stress on the stability of EVs.^37^ These factors can affect EVs structure and biological function and, subsequently, the outcomes reported in preclinical models.

Additionally, we observed different nomenclature used for EVs across all studies (Supplementary Table S1). While 85% (*n* = 11) of the studies reported the size of vesicles between 50 and 200 nm, the terms used to refer to the particles varied from exosome and exosome/ microvesicle (EMV) to nanovesicles, EV and sEV (small EV). According to the guidelines released by the international society for extracellular vesicles (ISEV) in 2018, the consensus recommendation on nomenclature is to use extracellular vesicles (EVs) as the generic term. Operational terms for EV subtypes have been proposed to distinguish them based on those specific characteristics. Based on this guideline, exosomes and microvesicles are collectively referred to as sEV ranging from 50 to 200 nm, unless the origin of the particle is explicitly mentioned.^36^ The differences in the formulation of the cell culture media, the incubation time before collecting the conditioned media, EV isolation methods and characterization techniques are considered as other potential sources of heterogeneity that can impose a more significant challenge to the translation of these findings. Reporting the amount of EV used in vivo or in vitro is one of these challenges that has been addressed in the 2018 MISEV update. In the 2018 update, normalizing the amount of EVs based on factors such as characteristics of isolated EVs, source characteristics or co-isolated standards is introduced as a strategy to overcome this challenge. In our meta-analysis, only one study reported a normalizing approach based on the number of cells cultured to isolate EVs. In terms of the therapeutic intervention, subgroup analysis showed no significant heterogeneity between the EV isolation and modification methods.

As a final consideration regarding EV treatment, EV biodistribution was investigated in 3 studies using diverse EV detection and tracking markers and imaging methods (Supplementary Table S6) highlighting the significant heterogeneity in current in vivo studies with widely varying tracking methodologies. While all 3 studies indicated the biodistribution of EVs within CNS in a timeframe of 2–4 hours following I.V. administration, 2 out of 3 studies additionally reported a more extensive distribution of EVs in the liver, lung, and spleen. These findings are aligned with the outcomes of a systematic review addressing the biodistribution of EVs post-administration in vivo.^38^ While understanding the biodistribution of EVs is crucial for their effective use as therapeutic biopharmaceuticals, the small number of studies (*n* = 3) investigating EV biodistribution restricts drawing definitive conclusions regarding the influence of administration routes on primary or secondary outcomes from the existing dataset.

### Limitations

While the majority of studies (*n* = 16) used intravenous administration, the comparative efficacy of each administration route cannot be determined due to different types of stem cells, EV vs CM, and various doses used across different studies. Furthermore, data was not sufficient to conduct a side-by-side comparison of different routes of administration.

The RoB assessment revealed a low outcome blinding (<25%) and unclear randomization level for all included studies. The lack of reporting information such as the randomization methods or sample size calculation was noted. Furthermore, the absence of critical information, not provided by the authors, such as image descriptions rather than quantitative data, not including the sex and age of the animals, and insufficient information about the immunization regimen, posed challenges for a robust meta-analysis. Of particular concern is the potential exclusion of negative findings from primary studies, which may lead to a bias favoring the reporting of positive outcomes. Such inherent bias poses a substantial challenge to achieving impartial and comprehensive conclusions.

Additionally, 18 out of 19 studies were conducted using small animals. This highlights the gap in translating these findings into human clinical trials, emphasizing the need for further research, especially involving larger animal subjects. Furthermore, outcome measures were highly varied, thus leading to insufficiently powered analyses of small groups in subgroups. All these factors contributed to the further complication in drawing definite conclusions.

## Conclusions and Future Directions

The data from the current meta-analysis indicates that stem cell-derived EV and CM have a notable impact on reducing disease severity, demyelination, and neuroinflammation in preclinical models of MS. Major limitations identified include the use of a limited number of MS animal models and inconsistent methods of analyses for secondary outcomes measures across studies employing the same model. These limitations underscore the necessity to standardize and improve the consistency of study methodologies and designs. Implementing such standardization would facilitate precise comparisons between studies and foster the generation of more dependable and robust conclusions.

Of note, the majority of the studies included were conducted on rodents, emphasizing the need for large animal studies to improve pre-clinical translation and treatment optimization prior to clinical trials. Furthermore, we identified the gaps in designing and conducting such preclinical studies. This information will inform future preclinical studies and could help facilitate the translation of stem cell-derived EV therapy as a potential therapeutic option for MS.

## Supplementary Material

szae011_suppl_Supplementary_Material

## Data Availability

The original contributions presented in the study are included in the article/ Supplementary Material. Further inquiries can be directed to the corresponding authors.
