# Supplementary material for: Stem Cell Derived Extracellular Vesicle Therapy for Multiple Sclerosis, A Systematic Review and Meta-Analysis of Preclinical Studies
Source: Stem Cells Transl Med. 2024 Mar 20;13(5):436–47. doi: 10.1093/stcltm/szae011 (PMC11092271; doi:10.1093/stcltm/szae011)
Supplement: szae011_suppl_Supplementary_Material [file szae011_suppl_supplementary_material.pdf]

Table 1. Summary of interventions in the included studies

Study	Cell type	Cell source	Cell modification	Intervention*	Administration's route/dose/frequency/time				
					Route	Dose/animal	Frequency	Time post induction	Particle size (ug)
**Riazifar**	BM-MSC	Human	Primed with IFN- γ	Exosome	I.V.	150 µg from 5-7 $*10^6$ cells or $1.06 \times 10^9 \pm 9.6 \times 10^7$ particles	B6: 1<br>Foxp3-eGFP": 2	B6: D18<br>Foxp3-eGFP: D12, D14	115
**Fathollahi**	AD-MSC	Mouse	-	sEV	I.N.	10 ug	13	D15 to D27 (Daily)	50 - 200
**Shamili**	BM-MSC	Mouse	Engineered EV	Exosome	I.V.	200 ug	3	Prophylactic: D12, D15, D18<br>Therapeutic: D12, D15, D18	Naïve EV: 100.8<br>EV with aptamer: 133 ± 15
**Li**	BM-MSC	Rat	-	Exosome	I.V.	100 ug (low)<br>400 ug (high)	1	D1	30 - 100
**Koohsari**	UC-MSC	Human	-	EV	I.V.	50 ug	1	D9	90.89
**Farinazzo**	AD-MSC	Mouse	-	Nanovesicle	I.V.	5 µg /300 ul PBS	Preventive: 3<br>Therapeutic: 3	Preventive: D3, D8, D13<br>Therapeutic: D12, D16, D20	Not reported
**Laso**	AD-MSC	Human	-	EV	I.V.	25 ug	1	D60	<100
**Jafarnia**	AD-MSC	Human	-	EV	I.V.	60 µg	1	D10	84.76
**Rajan TS (a)**	PDLSC	Healthy; RR-MS patients	-	EMVs	I.V.	25 ug	1	D14	Not reported

**Rajan TS (b)**	PDLSC	Healthy; RR-MS patients	-	EMVs	I.V.	25 ug	1	D14	Not reported
**Giunti**	BM - MSC	Mouse	primed with IFN-γ	sEV	I.V.<br>I.P.	The amount of s-EV corresponded to the supernatant from 10×10^6 (iv) or 3×10^6 (ip) cells	IV: 4	IV: alternate days for 8 days (D10 (onset) - D18)	30-100
							IP: 6	IP: daily for 6 days (D10(onset) - D16)	
**Zhang**	BM - MSC	Monkey	-	Exosome	I.V.	5×10^{10} particles	EAE: 7	EAE: twice a week for 4 wks. initiated on D10	30-150
							CPZ: 3	CPZ: 3 doses of EV on wks. 5 (CPZ ceased), 6 and 7	
**Bai**	BM-MSC	Human	-	CM	I.V.	500 ug	1	D18	-
**Wang**	BM - MSC	Mouse		CM	I.N.<br>I.V.<br>I.P.	60 ul	10	D3 - D12	-
**Giacoppo**	PDLSC	Humans	-	CM	I.V.	1000 ug	1	D14	-
**Yousefi**	AD-MSC	Mouse	-	CM	I.P.	1000 ul	4	D10, D17, D21, D28	-
**Sargent**	BM-MSC	Healthy; RR-MS patients	-	CM	I.V.	500 ug /100 ul	1	D16	-
**Shimojima**	DP-MSC	Human	-	CM	I.V.	3 ug /500 ul	1	D14	-
**Galeshi**	Ad-MSC	Human	-	CM	I.P.	2 ul	14	D14	-

I.V. intravenous; I.P. intraperitoneal; I.N. intranasal; EMVs: Exosome/Microvesicles; EV: Extracellular vesicle; CM; conditioned media.

*In the intervention column, EVs have been listed with the nomenclature used in each paper.

Table 2. EV characterisation methods in the included studies.

Study	Size / morphology		Membrane-associated proteins			Cytosolic Proteins recovered in EVs					Protein / RNA cargo	
	**Nanoparticle tracking**	**Microscopy**	**CD63**	**CD9**	**CD81**	**ALIX**	**TSG101**	**HSP70**	**Calnexin**	**Galectin-1**	**RNA seq**	**Proteomics**
**Riazifar**	NTA		FC	FC	WB<br>FC		WB	WB	WB	WB	Y	Y
**Fathollahi**	DLS	SEM	Dot-blot									
**Shamili**	DLS	AFM	FC<br>WB	FC								
**Li**		TEM	WB	WB		WB					Y	
**Koohsari**	DLS	SEM	FC	FC								
**Farinazzo**				WB			WB	WB				
**Laso**	NanoSight	EM	WB		WB	FM						
**Jafarnia**	DLS	EM	FC	FC								
**Rajan TS (a)**		FM	FC									
**Rajan TS (b)**												
**Giunti**		EM		WB		WB						
**Zhang**	qNano	TEM	WB			WB						

DLS: dynamic light scattering; EM: electron microscopy; NTA: nanoparticle tracking; SEM: scanning electron microscopy; AFM: atomic force microscopy; FM: fluorescence microscopy; FC: flowcytometry; WB: western blot.

Table 3. Secondary outcome of the included studies.

	CNS pathology				CNS repair (Remyelination / OPCs)	Inflammatory response		
	**Inflammation**	**Demyelination**	**Axonal damage**	**Astrogliosis**		**cytokines**	**Tregs**	**MOG-specific proliferation**
**Riazifar**	Iba-1 *↓	LFB *↓					Spleen NS<br>Lymph node NS<br>spinal cord (*↑ in IFNγ-primed group, NS in unprimed group)	
**Fathollahi**		LFB *↓				MOG recall response: IFN-γ, IL-17A, IL-10, TGF-β	Spleen *↑	
**Shamili**	H&E: NS except Exo-APT-PR (*↓)	LFB: NS except Exo-APT-PR (*↓)	Bielschowsky silver ↓axonal density (ND)<br><br>Toluidine blue *↑ total fibre number except Exo-PR		NG2 ↑ (ND)<br><br>G-ratio *↑ total fibre number except for Exo-PR	Serum/spleen:<br>IFN-γ, IL4, IL17 (NS)	Spleen: NS except Exo-APT-PR (*↑)	
**Li**	H&E: NS in low EV cons/*↓ in high EV cons)<br>CD68 (CNS) *↓<br>CD206 (CNS) *↑	LFB (NS in low EV cons and *↓ in high EV cons)				Serum:<br>TNF-α, IL-12 (*↓)<br>IL-10, TGF-β (*↑)		
**Koohsari**	H&E *↓	MBP ↑NS		GFAP ↓NS		Spleen:<br>IFNγ, TNFa, IL17a (*↓)<br>IL10, IL4 (*↑)	Spleen *↑	

**Farinazzo**	H&E *↓<br>Iba-1 *↓<br>CD3 *↓	Woelcke *↓<br>LFB *↓					Spleen/Lymph node (NS)	
**Laso**	H&E *↓<br>Iba-1 (brain) *↓<br>Iba-1 protein (spinal cord) NS	MBP *↑<br>CNPase *↑		GFAP (brain) *↓<br>GFAP (spinal cord) NS		Plasma:<br>IL-1β, IL-2, IL-4/IL-5, L-6, IL-12p70, IL-13, IL-17A, IL-18 (*↓)		
**Jafarnia**	H&E*↓	LFB *↓<br>MBP ↑			Olig2 (spinal cord) ↑NS		Spleen ↑NS	*↓
**Rajan TS (a)**	CD4 (*↓)	LFB (ND)	Golgi *↑ spinal density			CNS (EV & CM):<br>IL-17, IFN-γ, TNF-α, IL-6, IL-1β (*↓)<br>IL-10 *↑<br>Spleen (EV & CM):<br>IL-17, IFN-γ *↓<br>IL-10 *↑		CD4 (*↓)
**Rajan TS (b)**	H&E (no quantitative data)					CNS:<br>NALP3, active cleaved caspase 1, IL-1β, IL18, TLR4, NF-κB (*↓)<br>IκB-α *↑		H&E (no quantitative data)
**Giunti**						CNS:<br>TNF-α, IL-1β, IL6, Nos2 (*↓)		
**Zhang**	YM1 *↑<br>Iba-1 NS<br>iNOS *↓	MBP *↑	amyloidβ _precursor protein (App) *↓ (EAE)		EAE: NG2, APC *↑<br>CPZ: APC *↑<br>TEM: remyelination ↑ (ND)	CNS (EAE & CPZ):<br>TNF-α, IL-1β (*↓),<br>TGF-β (*↑)<br>IL-10 *↑(EAE)		
**Bai**		LFB ↓ (ND)				CNS:<br>IFN-γ, IL-17, TNF-α, IL-		

						2, IL-12p70 (*↓)<br>IL-10, IL-4 (*↑)		
**Wang**	CD4 / CD45 *↓<br>CD68, CD86 NS<br>Iba-1 *↓	LFB *↓				Serum:<br>TNF-α, IFNY, GM-CSF, IL-1β (*↓)	Lymph node*↑	
**Giacoppo**	H&E (no quantitative data)<br>CD4 *↓<br>CD68, iNOS *↓	MBP *↑	BNDF *↑<br>Cleaved Caspase 3 *↓					
**Yousefi**	H&E*↓					MOG recall response:<br>IFN-γ, IL17 (*↓)<br>IL4 (NS)	spleen *↑	*↓
**Sargent**	CD45/CD3 *↓ in naive CM group	Solochrome cyanine *↓ in naïve CM group only						*↓ in naïve and peak CM group
**Shimojima**	iNOS *↓<br>CD3 *↓<br>H&E (no quantitative data)<br>Arg-1 *↑	LFB *↓<br>Toluidine blue (ND)	SMI31 *↓			MOG recall response:<br>IL2, IFN-γ, IL17 *↓<br><br>CNS:<br>IFN-γ, IL-17, TNF-a, iNOS (*↓)<br>Arg-1, CD206 (*↑)		↓
**Galeshi**		Fluoromyelin*↓<br>MOG*↓		GFAP*↓	PDGFRα (NS)			

*↑ significant increase; *↓ significant decrease; NS: no significant difference; ND: No quantitative data.

BNDF: Brain-derived neurotrophic factor; SMI13: Neurofilament.

Table 4. Detailed scoring of additional ROB assessment criteria.

Study	Conflict of interest	Sample size calculation	Animal welfare licence or approved ethics	Blinding at any level	Randomisation at any level
**Riazifar**	Declared conflict of interest (Codiak and Velox Biosystems)	-	Stated	Blind clinical outcome assessment	Not reported
**Fathollahi**	Declare no conflict - funding reported	calculated by considering a 10% attrition	Stated	Not reported	Animal randomisation after EAE induction<br>Random histological evaluation
**Shamili**	Funding reported	-	Stated	Blind cell infiltration measurement<br>Blind quantification of remyelination and axonal injury	Animal randomisation after EAE induction<br>Randomisation in image analysis
**Li**	Declare no conflict - funding reported	-	Stated	Blind clinical outcome assessment	Animal randomisation before EAE induction and after EAE induction<br>Randomisation in EV imaging
**Koohsari**	Declare no conflict	-	Stated	Not reported	Animal randomisation after EAE induction
**Farinazzo**	Declare no conflict - funding reported	-	Stated	Blind clinical outcome assessment<br>Blind quantification of H&E and Iba-1 staining	Not reported
**Laso-García F**	Declare no conflict - funding reported	-	Stated	Not reported	Animal randomisation before disease induction
**Jafarnia**	Declare no conflict - funding reported	-	Stated	Not reported	-

**Rajan TS (a)**	Declare no conflict - funding reported	-	Stated	Not reported	Animal randomisation before EAE induction
**Rajan TS (b)**	Declare no conflict - funding reported	-	Stated	Not reported	Animal randomisation before EAE induction
**Giunti**	Declare no conflict	-	Stated	Not reported	Clinical outcomes randomly assessed
**Zhang**	Declare no conflict	determined based on the previous experiments	Stated	Blind assessment of all outcomes	Animal randomisation after EAE induction<br>Animal randomisation before Cuprizone diet<br>Random assignment of treatments
**Bai**	Declare no conflict - funding reported	determined based on power estimates	Stated	Blind histology assessment	Myelin quantification randomly measured
**Wang**	Declare no conflict - funding reported	-	Stated	Not reported	Not reported
**Giacoppo**	Declare no conflict	-	Stated	Not reported	Animal randomisation before EAE induction
**Yousefi**	Not reported	-	Stated	Blind clinical outcome assessment<br>Blind quantification of cell infiltration	Animal randomisation after EAE induction
**Sargent**	Not reported	-	Stated	Blind clinical outcome assessment<br>Blind histology assessments	Not reported
**Shimojima**	Declare no conflict - funding reported	-	Stated	Not reported	Random quantification of immunofluorescence staining
**Galeshi**	Declare no conflict	-	Stated	Not reported	Animal randomisation before LPC induction

Table 5. Immunisation protocol of studies using EAE animal model.

Study	Year	Mycobacterium Tuberculosis (mg/ml)	MOG_{35-55} (ug)	Pertussis toxin (ng/dose)	Days of Pertussis injection
**Riazifar**	2019[1]	4	C57BL/6J: 200<br>Reporter mice:100	C57BL/6J: 400<br>Reporter mice: 200	D0, D2
**Fathollahi**	2020[2]	-	150	-	D0, D1
**Shamili**	2019[3]	-	-	250	D0, D2
**Li**	2018[4]	10	1 g/ml guinea pig spinal cord + an equal volume of incomplete Freund's adjuvant		D0
**Koohsari**	2021[5]	0.4	400	250	D0, D2
**Farinazzo**	2018[6]	0.8	300	40	D0, D2
**Jafarnia**	2020[7]	0.4	400	250	D0, D2
**Rajan TS (a)**	2016[8]	1	300	500	D0, D2
**Rajan TS (b)**	2017[9]	1	300	500	D0, D2
**Giunti**	2021[11]	3	200	400	D0, D2
**Zhang**	2022[12]	4	200	200	D0, D2
**Bai**	2012[10]	-	200	500	D0, D2
**Wang**	2019[13]	-	(r)MOG: 100	300	D0, D2

			MOG_{35-55}: 200		
**Giacoppo**	2017[14]	1	300	500	D0, D2
**Yousefi**	2016[15]	4	200	300	D0, D2
**Sargent**	2017[16]	-	-	250	D0, D1
**Shimojima**	2016[17]	-	200	200	D0, D2

Table 6. EV biodistribution in the included studies

Study	Animal model	Tracking method and Markers	Imaging method	Imaging time post administration (hrs)	Biodistribution
**Riazifar[23]**	EAE	EV labelling using Lipophilic dye (DiR)	IVIS	3<br>24	Liver, lung, spleen, kidney, and brain
**Laso-García F[29]**	TMEV	TAPA1/CD81 staining as a positive marker in the brain.<br>EVs (CD63) colocalization with GFAP and NeuN	spectral confocal microscopy	2	Liver, lung, spleen, and brain
**Zhang Jing[34]**	Healthy	GFP transfected EVs (using CD63-GFP plasmid and electroporation) colocalization with OPCs	laser scanning confocal microscopy	4	CNS (other organs not studied)

IVIS: In Vivo Imaging System; GFAP: glial fibrillary acid protein; NeuN: neuronal specific nuclear protein; OPC: Oligodendrocyte Progenitor Cells.

Table 7. Subcategorised studies for meta-analysis.

Study	Subgroups
**Riazifar**	(a) Naïve stem cell derived EV in B6 mice<br>(b) IFNγ primed stem cell derived EV in B6 mice<br>(c) Naïve stem cell derived EV in FOXP3 reporter mice<br>(b) IFNγ primed stem cell derived EV in FOXP3 reporter mice
**Shamili**	(a) Exo-PR:prophylactic treatment with EV<br>(b) Exo-APT-PR:prophylactic treatment with EV conjugated with aptamer<br>(c) Exo-T:therapeutic treatment with EV<br>(d) Exo-APT-T:therapeutic treatment with EVconjugated with aptamer
**Li**	(a) Low EV dose<br>(b) High EV dose
**Farinazzo 2018**	(a) Before onset<br>(b) After onset
**Rajan 2016**	(a) Stem cell derived EV from MS patients<br>(b) Stem cell derived EV from healthy donors<br>(c) Stem cell derived CM from MS patients<br>(b) Stem cell derived CM from healthy donors
**Rajan 2017**	(a) Stem cell derived EV from MS patients<br>(b) Stem cell derived CM from MS patients
**Sargent**	(a) Naïve MSC-CM<br>(b) Peak EAE-MSC-CM<br>(c) Chronic EAE- MSC-CM
**Wang**	(a)(r)MOG induced EAE<br>(b) MOG $_{35\text{-}55}$ induced EAE

**Zhang 2022**	(a) EAE<br>(b) CPZ
**Giunti**	(a) IV<br>(b) IP

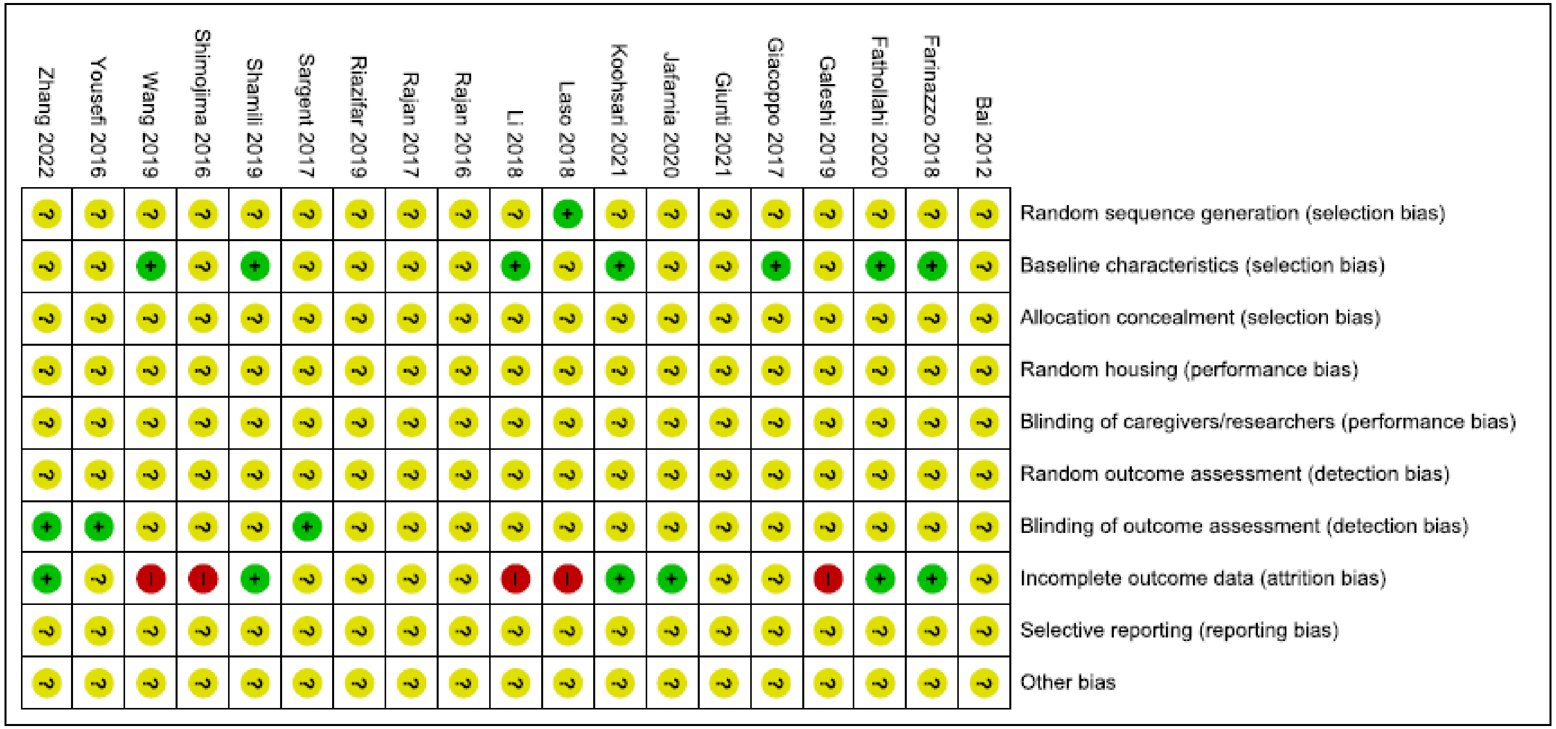

Figure 1. Risk of bias summary. Key: red, green, and yellow circles represent high, low, and unclear risk of bias.
